# Functional Electrical Stimulation of the Lateral Knee Muscles Can Reduce Peak Knee Adduction Moment during Stepping: A Pilot Study

**DOI:** 10.3390/bioengineering11090881

**Published:** 2024-08-30

**Authors:** Raziyeh Baghi, Gad Alon, Giovanni Oppizzi, Subham Badhyal, Peter Bowman, Li-Qun Zhang

**Affiliations:** 1 Department of Physical Therapy and Rehabilitation Science, University of Maryland, Baltimore, MD 21201, USA; rbaghi@som.umaryland.edu (R.B.); galon@som.umaryland.edu (G.A.); goppizzi@umd.edu (G.O.); pbowman@som.umaryland.edu (P.B.); 2 Department of Bioengineering, University of Maryland, College Park, MD 20742, USA; 3 Phoenix Children’s Hospital, Phoenix, AZ 85016, USA; subhambadhyal@gmail.com; 4 Department of Child Health, University of Arizona College of Medicine-Phoenix, Phoenix, AZ 85004, USA; 5 Department of Orthopaedics, University of Maryland, Baltimore, MD 21201, USA

**Keywords:** knee osteoarthritis, knee adduction moment, functional electrical stimulation, elliptical training

## Abstract

Knee osteoarthritis (KOA) is an age-dependent disease dominantly affected by mechanical loading. Balancing the forces acting on the medial knee compartment has been the focus of KOA interventions. This pilot study investigated the effects of functional electrical stimulation (FES) of the biceps femoris and lateral gastrocnemius on reducing peak knee adduction moment (pKAM) in healthy adults and individuals with medial KOA while stepping on an instrumented elliptical system. Sixteen healthy individuals and five individuals with medial KOA stepped on the robotic stepping system, which measured footplate-reaction forces/torques and ankle kinematics and calculated 3-D knee moments in real time using inverse dynamics. Participants performed four different tasks: regular stepping without FES as the baseline condition, stepping with continuous FES of the lateral gastrocnemius (FES_LG_), biceps femoris (FES_BF_), and simultaneous FES of both lateral gastrocnemius and biceps femoris (FES_LGBF_), throughout the elliptical cycle. The 3-D knee moments, tibia kinematics, and footplate-reaction forces were compared between the baseline and the three FES stepping conditions. Healthy participants demonstrated lower pKAM during each of the three FES conditions compared to baseline (FES_LG_ (*p* = 0.041), FES_BF_ (*p* = 0.049), FES_LGBF_ (*p* = 0.048)). Participants with KOA showed a trend of lower pKAM during FES, which was not statistically significant given the small sample available. Incorporating elliptical + FES as a training strategy is feasible and may help to enhance selective force generation of the targeted muscles and reduce the medial knee compartment loading.

## 1. Introduction

Knee osteoarthritis (KOA) has a complex etiology involving abnormal mechanical stress, inflammation, and trauma, which causes histological and biomechanical changes in the knee joint and surrounding structures [1,2]. Alterations in the normal knee kinematics and kinetics are among the main contributing factors to the development of the disease [3,4]. Moreover, a strong correlation exists between the increase in external knee adduction moment (KAM) and the disease progression as well as knee pain [5,6,7]. The direct contribution of KAM to increased contact force on the medial tibial plateau-medial femoral condyle surface compelled investigators to consider new treatment designs to minimize the KAM and thereby decrease the contact force on the medial compartment of the knee joint [3].

The literature provides strong evidence that both muscles and ligaments stabilize the knee joint. Both these active and passive components generate internal forces and moments that oppose external forces and moments acting on the knee [8,9,10]. Among muscles that significantly resist the external KAM during gait are the gastrocnemius (late stance) and biceps femoris (early stance) [11,12]. Targeted activation of these muscles may reduce the medial knee compartment loading by manipulating the internal forces.

Altering muscles’ force generation and strength is possible through functional electrical stimulation (FES), a specific use of neuromuscular electrical stimulation (NMES) that involves applying a train of electrical pulses to induce muscles’ contraction via surface electrodes during functional activities like walking. In vivo, NMES of the quadriceps components could significantly change the knee moments [13]. Therefore, targeted application of FES to invoke contractions of gastrocnemius and biceps femoris and generate an internal knee abduction moment may counteract the external knee adduction moment and diminish the load on the medial knee compartment.

Few studies have employed musculoskeletal modeling to investigate the effects of FES on KAM and medial compartment contact force during overground walking. Xu et al. simulated the impact of increased biceps femoris activation on knee joint loading and demonstrated that an additional activation of the biceps femoris during the stance phase of gait reduced the second peak of the medial knee joint contact force by 17% [14]. Similarly, Azmi et al. reported that a 20.8% increase in biceps femoris activation could effectively reduce the anterior shear force to zero and decrease the internal rotation torque by 188% [15]. These findings highlight the promising role of FES in reducing medial compartment contact force. However, these studies explored only overground walking and did not investigate the impact of muscle force redistribution in other functional exercises, such as stepping on an elliptical instrument.

Individuals with KOA are encouraged to participate in low-impact exercises suitable for lower limb muscle strengthening, such as elliptical training. Elliptical training induces smaller external KAM compared to overground walking and high-impact activities [16,17,18]. The smaller KAM during elliptical stepping may imply lower medial knee contact force during the stepping exercise. However, further manipulation of the muscle forces during stepping using FES may lead to higher internal forces that resist external KAM during overground walking. This pilot study investigated the effects of FES applied to the biceps femoris and lateral gastrocnemius on knee joint moments during elliptical stepping in healthy adults and individuals with KOA. We hypothesized that biceps femoris stimulation would reduce the peak knee adduction moment (pKAM) and peak knee internal rotation moment (pKIRM) while the lateral gastrocnemius stimulation would reduce the pKAM and increase the pKIRM. The hypothesis was based on the 3-D biomechanical determination of the functions of these muscles in controlling knee joint loading in the frontal and transverse planes [13,19,20].

## 2. Materials and Methods

### 2.1. Participants

A total of 21 participants, including 16 healthy adults and 5 individuals with medial KOA, participated in this study. The demographic characteristics of both groups are summarized in Table 1. The healthy participants had no history of pain or injury to the lower extremities. The inclusion criteria for the KOA group were the presence of pain in the medial compartment of the right knee more than one day per week in the last six weeks [21] and radiographic evidence of medial KOA with Kellgren and Lawrance (K/L) grade. The study’s exclusion criteria were as follows: severe cardiovascular disease, uncontrolled hypertension, presence of pain or injury to the hip or ankle that affects walking ability, and any pre-existing neurological condition. The study protocol was approved by the Institutional Review Board of the University of Maryland, Baltimore, and all participants provided written informed consent prior to enrollment.

### 2.2. Stepping System

**Figure 1:**
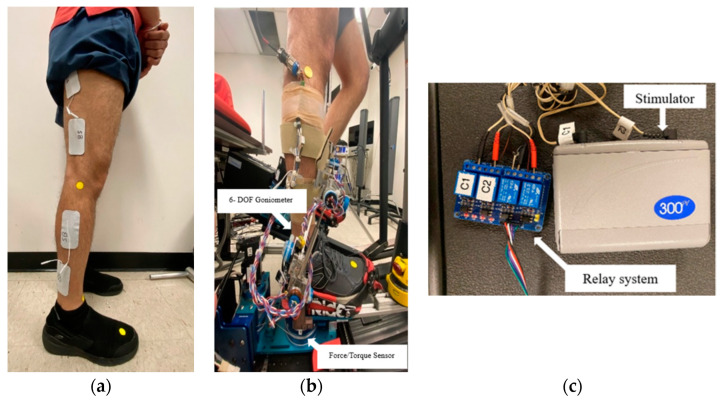
Electrode placement for the lateral gastrocnemius and biceps femoris. (**a**) The self-adhesive electrodes were placed on the muscles. The electrode locations were tested using an electrical stimulator to ensure the appropriate placement of the electrodes before the participant was positioned on the elliptical system; (**b**) fixation of the electrodes and goniometer on the participant’s leg during the elliptical exercise; (**c**) electrical stimulator and the customized synchronization relay system with four channels.

This study employed the robotic stepping system with real-time knee moment estimation described in [22,23,24]. Briefly, a six-degree-of-freedom (DOF) goniometer was used to measure the tibia kinematics. The proximal end of the goniometer was attached firmly to the anteromedial aspect of the tibia, and the distal end was connected to the footplate (Figure 1b). To measure the 3-D footplate reaction forces and torques, a six-axis force/torque sensor (JR3, Woodland, CA, USA) was mounted beneath each footplate (Figure 1b). One strap at the front and one on the back of the footplate was used to secure participants’ feet on the footplates during stepping (Figure 1a).

A standing position with 3-D zero tibial inclination angles, including anterior/posterior inclinations, medial/lateral inclinations, and internal/external rotation inclination, was measured on the stepping system. The following landmarks were aligned to measure the standing position: in the sagittal plane, the peripheral margin of the lateral tibial plateau, the lateral malleolus, and the center of the force/torque sensor were aligned, and in the frontal plane, the tibial tuberosity, the mid-point between the lateral and medial malleoli, the second metatarsal head, and the center of the force/torque sensor were aligned. Participants’ anthropometric data, tibia kinematics, and forces and moments measured at the footplate were used in a custom inverse dynamics algorithm to determine the knee moments in real time [24,25].

### 2.3. Experimental Procedure

Prior to electrode placement, the skin over the targeted muscles was prepared using alcohol pads to reduce electrical impedance and ensure full contact of the electrodes with the skin. Following skin preparation, 2 in × 3.5 in rectangle self-adhesive surface electrodes (Performa, Performance Health, Warrenville, IL, USA) were placed on the right limb’s lateral gastrocnemius and biceps femoris muscles, as illustrated in Figure 1b. Symmetric biphasic waveform with a frequency of 50 Hz, pulse duration (width) of 300 µs, and a peak current of up to 100 mA was delivered using a commercially available FES device (Empi 300 PV, Empi Inc., Clear Lake, SD, USA) (Figure 1c). To verify proper electrode placement and ensure adequate muscle activation, visible muscle contractions were observed upon initiation of the stimulation. In cases where muscle contractions were not evident at the participants’ maximum tolerable current intensity, the electrodes were repositioned to identify the best location for eliciting visible contractions.

After confirming proper electrode placement, participants were positioned on the elliptical trainer. The FES device was synchronized with the elliptical trainer’s cycle using a custom-built relay system, enabling the targeted delivery of stimulation during specific phases of the stepping motion. In a prior pilot attempt to fine-tune the testing protocol, we examined the effect of FES on pKAM with the stimulation being delivered during 0–35% of the elliptical cycle based on our repeated observations that pKAM usually happens during this phase of the cyclical stepping. However, using this strategy did not yield marked changes in the pKAM. Therefore, in the current study, we applied the FES continuously throughout the elliptical cycle to enhance these muscles’ activation, as used for a longer duration in a previous study [25]. The experimental protocol consisted of four conditions: (1) normal stepping without FES, (2) stepping with FES applied to the lateral gastrocnemius (FES_LG_), (3) stepping with FES applied to the biceps femoris (FES_BF_), and (4) stepping with FES applied simultaneously to both the lateral gastrocnemius and biceps femoris (FES_LGBF_). The sequence of applying the stimulation was random. For stepping + FES conditions, FES was applied to the muscles, with the stimulation initiated after the participants started stepping. The current intensity was gradually adjusted to the participant’s maximum tolerance level during the stepping motion. After completing each stepping condition, participants were given a 2 min rest period.

### 2.4. Data Processing

Data from 10 consecutive stepping cycles were selected for analysis for each condition. A cycle was identified by the right foot’s motion from its foremost position back to the same point. The initial 50% of the stepping cycle, representing the loading stance, was specifically analyzed. During this phase, the external knee moment (EKM) components, namely, the knee flexion moment (KFM), knee adduction moment (KAM), and knee internal rotation moment (KIRM), were normalized to each participant’s body weight and height and expressed as a percentage of the product (% (BW × HT)). The knee adduction moment impulse (ImpKAM) was determined by integrating the KAM over the loading stance phase. These EKM components were further normalized across the entire stepping cycle duration and interpolated to represent a full 100% cycle range. The peak value of each EKM component during the loading phase was determined. Additional calculated parameters include the tibia inclination angles (anterior, medial, and internal rotation) and footplate-reaction forces (lateral, anterior, and vertical) at the time of pKAM. Averages of pKFM, pKAM, pKIRM, ImpKAM, and tibia inclination angles and footplate-reaction forces corresponding to the pKAM were calculated from these ten cycles for each participant. In general, the term electrical charge (Q) is calculated as the product of peak current (I) and the time (t) of the applied current (Q = I × t). Therefore, to quantify the electrical stimulation delivered to each muscle in this study, the total electrical charge (Q) received by the participants during the stimulation period was calculated using Equation (1). With the balanced, symmetrical biphasic stimulation pulses, the net charge is zero. Here, we calculate the absolute amount of charges, i.e., |Q|.

|Q| = PD × 2 × I × F × SD(1)

where:

|Q| = the absolute amount of electrical charge, measured in coulomb (C).
PD = pulse duration (s).
2 = having 2 opposing pulses in a single par of stimulation pulses.
I = intensity or peak current amplitude, measured in ampere (A).
F = frequency, measured in Hertz (Hz).
SD = stimulation duration during the whole 10 stepping cycles, measured in seconds (s) (the total time that muscle received FES during stepping).

### 2.5. Statistical Analysis

Statistical analyses were performed using a paired *t*-test with Bonferroni correction to compare pKAM, ImpKAM, pKFM, pKIRM, tibia inclination angles, and footplate-reaction forces corresponding to the pKAM between each of the FES conditions (FES_LG_, FES_BF_, and FES_LGBF_) and the baseline condition (BASELINE). The Bonferroni correction was applied to adjust for multiple comparisons, with the significance level set at α = 0.05. Due to the small sample size of the KOA group, a non-parametric test, the Wilcoxon Signed Rank Test, with Bonferroni adjustment, was used to compare each outcome measure between the three FES conditions and the baseline condition. An independent samples *t*-test was used to compare electrical charge and FES intensity between the KOA group and healthy participants. Before conducting the independent samples *t*-test, Levene’s test was used to assess the equality of variances between the two groups [26].

## 3. Results

### 3.1. Electrical Charge and Intensity

The demographic characteristics of the study participants are presented in Table 1. Lateral gastrocnemius stimulation intensity (mean ± standard deviation) was 43.67 ± 24.21 mA for the healthy volunteer’s group, and 43.50 ± 27.58 mA for the KOA group. The stimulation intensity of the biceps femoris was 39.92 ± 20.07 mA for the healthy group and 55.50 ± 41.72 mA for the KOA group. Regarding the total electrical charge, both groups received similar electrical charges during the FES_LG_ (105.25 ± 118.68 mC for the healthy group and 56.85 ± 40.85 mC for the KOA group). The electrical charge for the biceps femoris was likewise not significantly different between the groups (104.48 ± 115.53 mC for the healthy group and 56.85 ± 40.85 mC for the KOA group). The large standard deviation indicated diverse responses to the stimulation among the participants.

### 3.2. FES Impact on Stepping Characteristics in the Healthy Group

**Figure 2:**
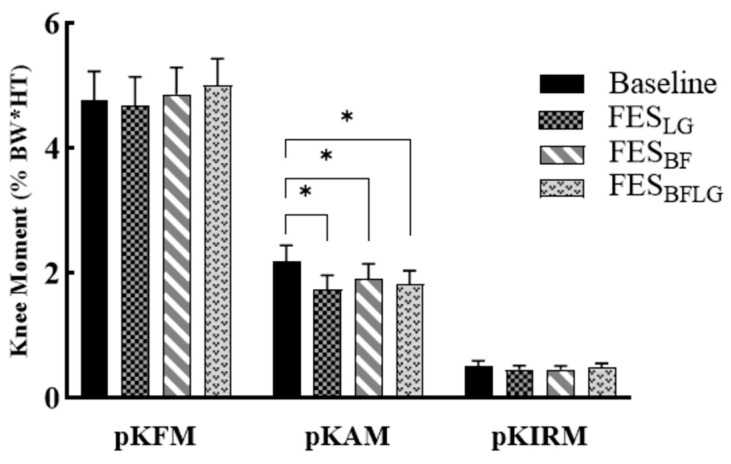
Peak knee flexion moment (pKFM), peak knee adduction moment (pKAM), and peak internal rotation moment (pKIRM) during stepping without FES (BASELINE), with FES of lateral gastrocnemius (FES_LG_), with FES of biceps femoris (FES_BF_), and FES of both lateral gastrocnemius and biceps femoris (FES_LGBF_) in the healthy group (n = 16). Error bars represent the standard error. * *p* < 0.05.

**Figure 3:**
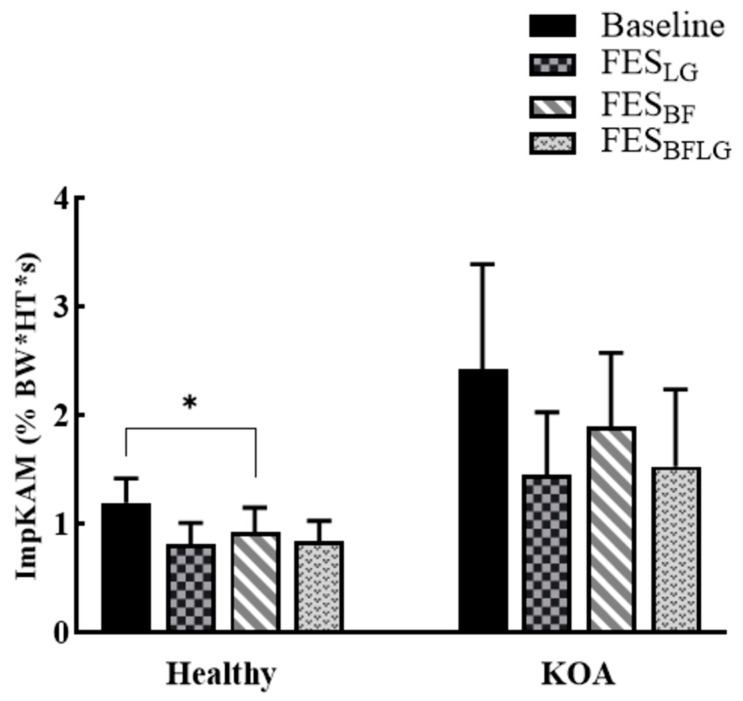
Knee adduction moment impulse (ImpKAM) during baseline and FES conditions in the healthy and KOA groups during stepping without any FES (BASELINE), with FES of lateral gastrocnemius (FES_LG_), with FES of biceps femoris (FES_BF_), and with FES of both lateral gastrocnemius and biceps femoris (FES_LGBF_) in the healthy group (n = 16) and the KOA group (n = 5). Error bars represent the standard error. * indicates significant difference with *p* < 0.05.

In the healthy group, all three FES conditions (FES_LG_, FES_BF_, and FES_LGBF_) led to significantly lower pKAM compared to the baseline condition (BASELINE) (Figure 2). The *p*-values for the comparisons were as follows: FES_LG_ vs. BASELINE (*p* = 0.041), FES_BF_ vs. BASELINE (*p* = 0.049), and FES_LGBF_ vs. BASELINE (*p* = 0.048) (Table 2). In contrast, pKFM and pKIRM did not change statistically, as seen in Figure 2. Additionally, the FES_BF_ condition resulted in a significantly lower ImpKAM than the BASELINE condition (*p* = 0.033) (Figure 3).

### 3.3. FES Impact on Stepping Characteristics in the KOA Group

**Figure 4:**
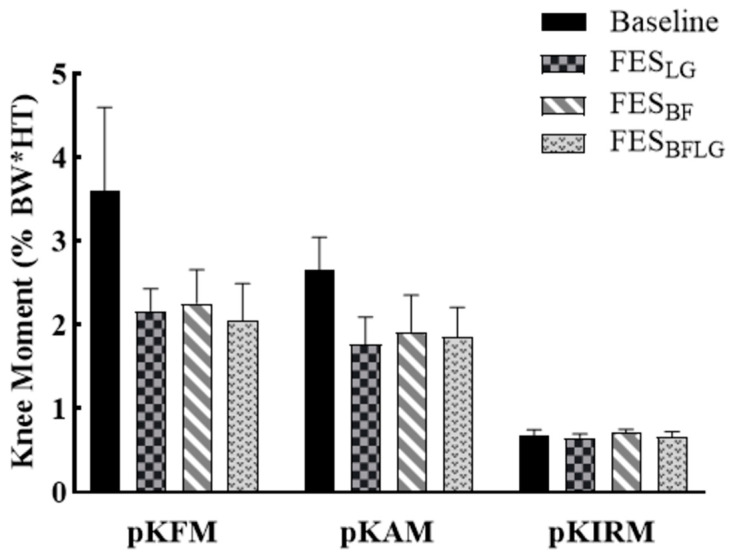
The peak knee flexion moment (pKFM), peak knee adduction moment (pKAM), and peak internal rotation moment (pKIRM) during stepping without any FES (BASELINE), with FES of lateral gastrocnemius (FES_LG_), with FES of biceps femoris (FES_BF_), and with FES of both lateral gastrocnemius and biceps femoris (FES_LGBF_) in the KOA group (n = 5). Error bars represent the standard error.

The results of the KOA group were considerably different from that of the Healthy group. The KOA group did not show any significant difference in the KAM between each of the FES conditions and the baseline (*p* = 0.240 for FES_LG_, *p* = 0.129 for FES_BF_, and *p* = 0.068 for FES_LGBF_), given the small KOA sample available (Figure 4). Similarly, there was no difference in the ImpKAM between each of the FES conditions and the baseline (*p* = 0.414 for FES_LG_, *p* = 0.675 for FES_BF_, and *p* = 0.204 for FES_LGBF_) (Figure 3, Table 3).

## 4. Discussion

The present study investigated the immediate effect of FES applied to the lateral gastrocnemius and biceps femoris muscles on knee joint moments during elliptical stepping. Due to the small sample of patients with OA (n = 5), we presented and accordingly discussed the findings separately. The data obtained from healthy volunteers supported our first hypothesis and revealed that continuous stimulation of each muscle during elliptical stepping, including concurrent stimulation of muscles above and below the knee, significantly reduced the pKAM. The corresponding reductions were 45% with FES_LG_, 28% with FES_BF_, and 37% with FES of both muscles combined. Furthermore, stimulation of the biceps femoris alone led to a 27% reduction in the ImpKAM. However, we did not find any change in the pKIRM following stimulation of the lateral gastrocnemius or biceps femoris muscles. These results suggest that FES can effectively reduce KAM during functional activities and exercises.

### 4.1. Healthy Participants’ Response to FES

The demonstrated efficacy of FES in reducing pKAM is consistent with previous research that has reported lower KAM and knee compressive forces during level walking with stimulation of the long head of biceps femoris as shown by musculoskeletal modeling [14,15]. The distal attachment of the biceps femoris to the fibular head, when activated through neuromuscular electrical stimulation, may result in the shortening of the muscle–tendon unit. This contraction generates a laterally directed force on the fibula, leading to an abduction moment at the knee joint. Similarly, the stimulation of the lateral gastrocnemius muscle generates a force vector oriented from the muscle’s proximal attachment on the lateral femoral condyle towards its distal attachment on the calcaneus. The lateral component of this force vector creates an abduction moment at the knee joint. Consequently, the stimulation of the biceps femoris and lateral gastrocnemius has the potential to reduce the knee adduction moment by counteracting the external adduction moment acting on the knee during weight-bearing or functional activities such as walking. These findings are consistent with previous research demonstrating that the biceps femoris and lateral gastrocnemius have valgus moment arms and generate valgus moments in response to the external varus moment [27,28].

Contrary to our findings, Xu et al. did not observe any reduction in the pKAM by stimulation of the lateral gastrocnemius. One explanation is that the functional activity assessed in their study, overground walking, differed from the elliptical stepping explored in our study. In the above-mentioned study, manually triggered FES of the muscles by participants during the stance phase of gait was implemented, while participants in our study were not in control of the triggering FES, and advanced technology was used to control the FES throughout the whole elliptical cycle automatically. These differences between the two methodologies may explain the non-congruent results. Furthermore, electromyographic studies have shown that the gastrocnemius muscle exhibits significantly lower mean and peak EMG activity during elliptical stepping compared to overground and treadmill walking in healthy adults [29,30]. Conceivably, the application of neuromuscular electrical stimulation to the gastrocnemius during elliptical stepping may activate the type II muscle fibers to a greater extent than during voluntary contractions, thereby exerting a more pronounced effect on knee joint loading. In the current study, the FES was applied throughout the entire elliptical cycle, whereas in the previous studies, stimulation was limited to the stance phase of gait [14,15]. The continuous muscle stimulation may enhance its activation and further reduce medial knee compartment loading.

Our findings did not reveal any significant differences in pKFM, pKIRM, tibia kinematics, or footplate-reaction forces when applying the FES during elliptical stepping, but we did observe a reduction in the pKIRM and an increase in the tibia internal rotation during the FES conditions compared to the BASELINE. Azmi et al. reported a reduction in peak tibia internal rotation torque following FES of the long head of the biceps femoris during the stance phase of overground walking [15], while we found a (nonsignificant) trend of such a reduction in the pKIRM during FES of biceps femoris compared to the BASELINE. Methodological differences between the two studies may provide a plausible explanation. Azmi et al. computed the knee internal rotation torque that occurred at the time of peak anterior shear force, while our study reported the peak internal rotation moment that occurred during the first 50% of the elliptical cycle (loading phase). Moreover, Burnfield et al. reported that elliptical stepping generally involved higher biceps femoris activities than that during the stance phase of overground walking [29,30]. Accordingly, FES-induced change in pKIRM may be less evident and thus not significant. Similarly, Xu et al. found that FES applied to the long head of the biceps femoris during overground walking reduced the second peak of medial knee joint loading by up to 0.17 body weight. In their study, the second peak knee adduction moment (pKAM) was significantly reduced, while pKFM, kinematics, and ground reaction forces (GRF) remained unchanged with FES, which is consistent with our findings. In the present study, the stepping speed did not change during the application of FES. Consequently, we did not observe any changes in footplate-reaction forces during the three FES conditions, as footplate-reaction forces, similar to GRF, are related to stepping speed. This observation also suggests that the unilateral application of FES during stepping did not cause any asymmetry in stepping patterns between the two sides.

### 4.2. KOA Group Response to FES

The results obtained from the five patients with KOA inform us of the feasibility of using a hybrid FES and elliptical in this patient population. All tolerated well both the elaborate testing procedure and the application of the FES. The tolerance of the stimulation intensity varied considerably among the five participants, as reflected in a very large standard deviation reported in the results section. Unlike healthy participants, the ones with KOA had complained of knee pain and, based on the literature, likely had weakness in the muscles surrounding the knee, most likely making them less excitable [31,32]. As a result, we were able to activate only a portion of biceps femoris and lateral gastrocnemius motor units, thereby minimizing the pKAM but insufficiently reaching a statistical or clinical conclusion. Additionally, the physical activity level of patients with KOA is known to be lower than that of healthy participants. This difference in physical activity capacity may affect participant’s tolerance of the FES intensity and, consequently, their response to it. The amount of stimulation received by the KOA participants was relatively low compared to the healthy volunteers in our investigation. Future studies will require modifications of the FES parameters aiming to induce strong muscle contractions, incorporate conditioning sessions to increase tolerance to the stimulation [33], and potentially add additional muscle groups based on 3-D actions of targeted muscles [13,19,20].

The current study has several limitations that should be acknowledged. First, due to the small sample size of the KOA group, we refrained from generalizing the findings of this study to individuals with KOA. Considering an effect size of 0.8, α = 0.05, and a power of 80%, we will need a sample size of 15 participants with KOA in future studies to observe any potential effect of FES on knee loading. Individuals with KOA may exhibit altered muscle activation patterns during functional activities, such as increased knee muscle co-contraction [34,35], and they may respond differently to the FES of the lateral knee muscles. A larger sample may contribute to identifying responders vs. non-responders and the ability to personalize the responses to the FES between different individuals. In addition, we did not assess the effects of FES on other major muscles that may counteract the external KAM, such as the vastus lateralis, gluteus medius, and tensor facia latae. We suggest exploring the effect of FES of these other important muscles on the knee loading and potentially assessing which muscle may contribute to further augmenting pKAM reduction during elliptical stepping. Furthermore, detailed testing of knee integrity and functionality, including 3-D knee stability, muscle strength, and perceived pain, will strengthen the experimental design. Finally, the current study only investigated the acute effects of FES on pKAM. Further research is needed to evaluate the long-term effects of rehabilitation programs incorporating FES on knee joint loading in individuals with KOA.

## 5. Conclusions

Functional electrical stimulation may emerge as a new technology option for reducing medial knee loading. With further studies of a larger sample size, it is plausible to project that the combination of FES and elliptical stepping as a hybrid strategy may help clinicians to offer a treatment for patients with KOA and may boost the effect of either or both technologies in reducing the pKAM.

## Figures and Tables

**Table 1:** Participant descriptive characteristics.

Characteristics	KOA (n = 5)	Healthy Control (n = 16)
Age (years)	63.19 (9.57)	43.82 (14.39)
Height (m)	1.70 (0.08)	1.73 (0.07)
Mass (kg)	69.96 (26.46)	75.66 (18.92)
BMI (kg·m^−1^)	28.28 (4.10)	25.07 (5.69)
Gender	Female: 3, Male: 2	Female: 7, Male: 9

KOA: knee osteoarthritis; BMI: body mass index.

**Table 2:** Mean (standard deviation) of knee moments, tibia inclination angles, footplate reaction forces, and stepping speed during stepping with BASELINE, FES_LG_, FES_BF_, and FES_LGBF_ in the healthy group (n = 16).

	BASELINE	FES_LG_	FES_BF_	FES_LGBF_
Speed (rpm)	32.17 (4.67)	30.08 (5.78)	30.30 (5.48)	30.56 (6.97)
pKFM (%(BW×HT))	4.77 (1.86)	4.69 (1.83)	4.87 (1.71)	5.01 (1.64)
pKAM (%(BW×HT))	2.19 (1.02)	1.74 (0.92) *	1.91 (0.97) *	1.82 (0.85) *
pKIRM (%(BW×HT))	0.52 (0.30)	0.46 (0.25)	0.46 (0.24)	0.49 (0.26)
ImpKAM (%(BW×HT×s))	1.20 (0.89)	0.81 (0.80)	0.93 (0.88) *	0.84 (0.74)
Anterior tibia inclination (deg)	7.28 (5.61)	8.61 (7.70)	9.20 (6.85)	9.53 (8.47)
Medial tibia inclination (deg)	−7.44 (3.61)	−5.32 (4.17)	−6.69 (3.72)	−8.24 (2.18)
Internal tibia rotation inclination (deg)	−4.48 (6.32)	−4.18 (5.96)	−3.96 (6.39)	−3.92 (6.11)
Lateral footplate reaction force (% BW)	−0.69 (2.77)	−0.46 (2.03)	−0.60 (2.74)	−0.67 (2.80)
Anterior footplate reaction force (% BW)	−8.34 (6.87)	−7.98 (6.52)	−7.51 (6.46)	−7.70 (7.04)
Vertical footplate reaction force (% BW)	77.26 (13.22)	79.06 (13.05)	79.09 (12.36)	77.56 (13.17)

* *p* < 0.05; FES_LG_: functional electrical stimulation of lateral gastrocnemius; FES_BF_: functional electrical stimulation of biceps femoris; FES_LGBF_: functional electrical stimulation of both lateral gastrocnemius and biceps femoris.

**Table 3:** Mean (standard deviation) of knee moments, tibia inclination angles, footplate reaction forces, and stepping speed during stepping with BASELINE, FES_LG_, FES_BF_, and FES_LGBF_ in the KOA group (n = 5).

	BASELINE	FES_LG_	FES_BF_	FES_LGBF_
Speed (rpm)	33.51 (4.62)	34.75 (5.96)	35.07 (6.62)	35.38 (6.77)
pKFM (%(BW×HT))	3.60 (2.24)	2.17 (0.58)	2.26 (0.90)	2.05 (0.89)
pKAM (%(BW×HT))	2.65 (0.89)	1.77 (0.72)	1.92 (0.98)	1.85 (0.72)
pKIRM (%(BW×HT))	0.68 (0.15)	0.65 (0.11)	0.71 (0.09)	0.66 (0.13)
ImpKAM (%(BW×HT×s))	2.43 (2.16)	1.46 (1.28)	1.90 (1.52)	1.53 (1.42)
Anterior tibia inclination (deg)	9.23 (7.38)	5.34 (11.24)	7.25 (9.89)	8.52 (7.23)
Medial tibia inclination (deg)	−5.29 (4.41)	−4.62 (4.13)	−4.95 (4.10)	−4.85 (4.07)
Internal tibia rotation inclination (deg)	−3.78 (2.55)	−2.19 (4.27)	−1.74 (3.95)	−2.65 (2.87)
Lateral footplate reaction force (% BW)	−1.13 (1.10)	−0.45 (2.15)	−0.63 (2.59)	1.22 (1.08)
Anterior footplate reaction force (% BW)	−4.82 (4.34)	−4.59 (5.35)	−3.29 (2.89)	−2.87 (1.70)
Vertical footplate reaction force (% BW)	79.50 (6.33)	78.99 (11.24)	77.29 (12.40)	80.85 (13.13)

FES_LG_: functional electrical stimulation of lateral gastrocnemius; FES_BF_: functional electrical stimulation of biceps femoris; FES_LGBF_: functional electrical stimulation of both lateral gastrocnemius and biceps femoris.

## Data Availability

The datasets presented in this article are not readily available because they are part of an ongoing study. Requests to access the datasets were directed to the corresponding authors’ e-mail addresses.
